# Resistin Increases Monolayer Permeability of Human Coronary Artery Endothelial Cells

**DOI:** 10.1371/journal.pone.0084576

**Published:** 2013-12-27

**Authors:** Md Saha Jamaluddin, Shaoyu Yan, Jianming Lü, Zhengdong Liang, Qizhi Yao, Changyi Chen

**Affiliations:** Molecular Surgeon Research Center, Division of Surgical Research, Michael E. DeBakey Department of Surgery, Baylor College of Medicine, Houston, Texas, United States of American; UAE University, Faculty of Medicine & Health Sciences, United Arab Emirates

## Abstract

Resistin has been linked to obesity, insulin resistance, atherosclerosis, and the development of cardiovascular disease. Nevertheless, the effects and the molecular mechanisms of resistin on endothelial permeability, a key event in the development of atherosclerosis, inflammation, and vascular disease, are largely unknown. In order to determine the effect of resistin on endothelial permeability, human coronary artery endothelial cells (HCAECs) were treated with clinically relevant concentrations of resistin and the endothelial permeability was measured using the Transwell system with a Texas-Red-labeled dextran tracer. The permeability of HCAEC monolayers treated with resistin (80 ng/mL) was 51% higher than the permeability of control monolayers (*P*<0.05). The mRNA levels of tight junction proteins zonula occludens-1 (ZO-1) and occludin in resistin-treated cells were 37% and 42% lower, respectively, than the corresponding levels in untreated cells. The protein levels of these molecules in resistin-treated cells were significantly reduced by 35% and 37%, respectively (*P*<0.05), as shown by flow cytometry and Western blot analysis. Superoxide dismutase (SOD) mimetic MnTBAP effectively blocked the resistin-mediated reduction of ZO-1 and occludin levels in HCAECs. In addition, superoxide anion production was increased from 21% (untreated cells) to 55% (cells treated with 40 ng/mL resistin), and 64% (resistin, 80 mg/mL) (*P*<0.05). The natural antioxidant Ginkgolide A effectively inhibited resistin-induced increase in permeability and the increase in superoxide anion production in HCAECs. Furthermore, resistin treatment significantly activated p38 MAPK, but not ERK1/2. Pretreatment of HCAECs with a p38 inhibitor effectively blocked resistin-induced permeability. These results provide new evidence that resistin may contribute to the vascular lesion formation via increasing endothelial permeability through the mechanism of oxidative stress and the activation of p38 MAPK.

## Introduction

Resistin is an adipokine that was discovered in 2001 and named for its resistance to insulin action. Resistin is also known as adipocyte-secreted factor (ADSF) or FIZZ3 (found in inflammatory zone 3) [1]–[4]. The serum concentration of resistin in humans ranges from 7 to 22 ng/mL; however, in patients with type 1 or 2 diabetes, obesity, and/or inflammatory conditions, plasma resistin levels may exceed 40 ng/mL [5]. The major cell populations that express and produce resistin in humans are peripheral blood mononuclear cell (PBMC), macrophages, and bone marrow cells [6]–[8]. Resistin has been associated with inflammatory markers, coronary artery disease, ovarian epithelial carcinoma, and cardiovascular disease (CVD) in the metabolic syndrome [9]–[13].

Vascular inflammation and increased endothelial permeability are critical factors causing atherosclerosis and several other diseases [14]–[17]. Endothelial barrier function is maintained by the tight junction molecules such as ZO-1 and occludin and adherens junction molecules such as VE-cadherin [18]–[20]. Several cytokines and growth factors, like tumor necrosis factor-α (TNF-α), interleukin-1β, and platelet-derived growth factor, can disrupt these junctional molecules and increase endothelial permeability [21], [22]. Mitogen-activated protein kinases (MAPKs) and other signal molecules are involved in the regulation of endothelial permeability [23]–[26]. In this study, we examined the effect and potential molecular pathways of resistin on endothelial permeability in human coronary artery endothelial cells (HCAECs). Specifically, monolayer endothelial permeability, the expression of specific endothelial junction molecules, oxidative stress, and MAPK signal transduction molecules are investigated.

## Materials and Methods

### Chemicals and Reagents

Human recombinant resistin was purchased from Peprotech (Rocky Hill, NJ, USA). Horseradish peroxidase-conjugated goat anti-rabbit IgG and anti-mouse IgG were purchased from Santa Cruz Biotechnology (Santa Cruz, CA). The iQ SYBR Green Supermix, Bio-Plex Phosphoprotein Detection Reagent Kit, and total target kit for Bio-Plex luminoassay were obtained from Bio-Rad Laboratories (Hercules, CA). Ginkgolide A was obtained from LKT laboratories (St. Paul, MN). MnTBAP [Mn(III)tetrakis(4-benzoic acid) porphyrin Chloride] was purchased from Calbiochem (La Jolla, CA). Antibodies against human TLR4, TLR2, p38, phosphorylated p38, JNK, phosphorylated JNK, ERK1/2, phosphorylated ERK1/2 and CD144 (VE-cadherin) were purchased from Cell Signaling Technology, Inc. (Danvers, MA). Mouse anti-zonula occluden-1 (ZO-1) and rabbit anti-occludin with or without FITC-conjugated antibodies were purchased from ZYMED (South San Francisco, CA). All other chemicals and reagents were obtained from Sigma (St Louis, Mo), unless otherwise stated.

### Endothelial Permeability

HCAECs were obtained from Gelantis (San Diego, CA) and were cultured in HCAEC growth medium (Gelantis). Paracellular permeability was studied in a Coaster Transwell system as previously described [27]. Briefly, fully confluent HCAEC monolayers were treated with different concentrations of resistin (20, 40, and 80 ng/mL), with or without pre-treatment with Ginkgolide A (5 µM) for 30 minutes. In separate experiments, cells were treated with either TNF-α (2 ng/mL) or resistin (80 ng/mL) for 24 hours, in the presence or absence of Ginkgolide A. To determine the involvement of MAPKs, cells were incubated with specific inhibitors of p38 (SB203580, 10 µM), ERK1/2 (PD98059, 50 µM) or JNK (SP600125, 25 µM) for 30 minutes, followed by incubation with resistin (80 ng/mL) for 24 hours. Equal amounts of Texas-Red-labeled dextran tracer were added to the upper chamber of the Transwell system in all the experiments. The amount of tracer penetrating through the cell monolayer into the lower chamber was measured using a fluorometer. The permeability index was calculated using the tracer’s concentration in the lower and in the upper chambers.

### Real-time RT-PCR

HCAEC monolayers were incubated with different concentrations of resistin (20, 40, and 80 ng/mL) for 24 hours. Total RNA extraction and cDNA reverse transcription were performed as previously described [28]. The primers for VE-cadherin, ZO-1, and occludin are described in our previous publication [29]. The iQ SYBR green Supermix Kit and iCycler iQ Real-time PCR detection system (Bio-Rad) were used for real-time PCR reaction. Sample cycle threshold (Ct) values were determined. Expression for each target gene in each sample was normalized to β-actin. Ct values were calculated as [ΔCt = 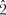
(Ct β-actin–Ct gene of interest)].

### Western Blot

Confluent HCAEC monolayers were incubated with resistin (80 ng/mL), in the presence or absence of MnTBAP (2 µM), for 24 hours. Proteins were extracted from the cells using cell lysis buffer. Equal amounts of protein (40 µg) were loaded onto 10% SDS-PAGE, fractionated by electrophoresis, and transferred to nitrocellulose membranes (Bio-Rad). The membranes were incubated with the primary antibody at 4°C overnight. Primary antibodies against ZO-1 and occludin-1 were used at a dilution of 1∶2000. The primary antibody for VE-Cadherin was used at a dilution of 1∶10000, and the β-actin antibody was used at a dilution of 1∶20000 (Millipore, Billerica, MA). The membranes were then incubated with secondary anti-rabbit (1∶5000) or anti-mouse (1∶10,000) horseradish peroxidase-labeled antibodies for 45 minutes at room temperature. Bands were visualized using Pierce ECL Western Blotting Substrate (Thermo Scientific, Rockford, IL). The band density was analyzed by ImageJ (1.47) software (NIH). For MAPKs, HCAECs were incubated with or without resistin, or pretreated with MnTBAP before treating them with resistin (80 ng/mL) for 45 minutes. The protein levels of MAPK were determined by Western Blot. Protein levels of TLR4 and TLR2 in HCAEC and EA.hy926 cells were also confirmed by Western blot.

### Flow Cytometry

Flow cytometry analysis was performed as previously described [29]. To measure reactive oxygen species (ROS) production, cultured HCAEC monolayers were incubated with different concentrations (40 or 80 ng/mL) of resistin, or pre-incubated with Ginkgolide A (5 µM) for 30 minutes, followed by resistin treatment (80 ng/mL) for 24 hours. One mL of dihydroethidium (DHE) (3 µM) was added into each well of the six-well plates and the cells were incubated for another 20 minutes at room temperature. Cells were fixed with 2% paraformaldehyde for 15 minutes and analyzed using FACSCalibur flow cytometer (Becton Dickinson, San Jose, CA). To detect the expression level of junction molecules, HCAEC monolayers were incubated with 40 ng/mL of resistin, with or without Ginkgolide A, for 24 hours. Cells were incubated with FITC-conjugated antibodies against VE-cadherin, ZO-1 or occludin, and then fixed with 2% paraformadehyde for 15 minutes. Cells stained for each junction molecule were detected and analyzed by flow cytometry.

### Bio-Plex Luminex Immunoassay

HCAECs were incubated with resistin (80 ng/mL) for 0, 5, 10, 20, 30, 45, 60, and 90 minutes. Cell lysates were collected using a cell lysis buffer. For each time point, MAPK was detected in a 30 µg protein sample. Bio-Plex phosphoprotein and total target assay kit was used on a Luminex multiplex system (Bio-Rad), according to the manufacturer’s instructions. Phosphorylated and total MAPK (ERK1/2, p38 and JNK) proteins were quantified and the results are presented as the ratio of phosphorylated versus total target proteins. The assay was performed in duplicate.

### Statistical Analysis

Data are expressed as the mean ± SD. Comparisons were made using the Student’s t-test. A *P* value <0.05 was considered statistically significant.

## Results

### Resistin Increases Permeability in HCAEC Monolayers

**Figure 1:**
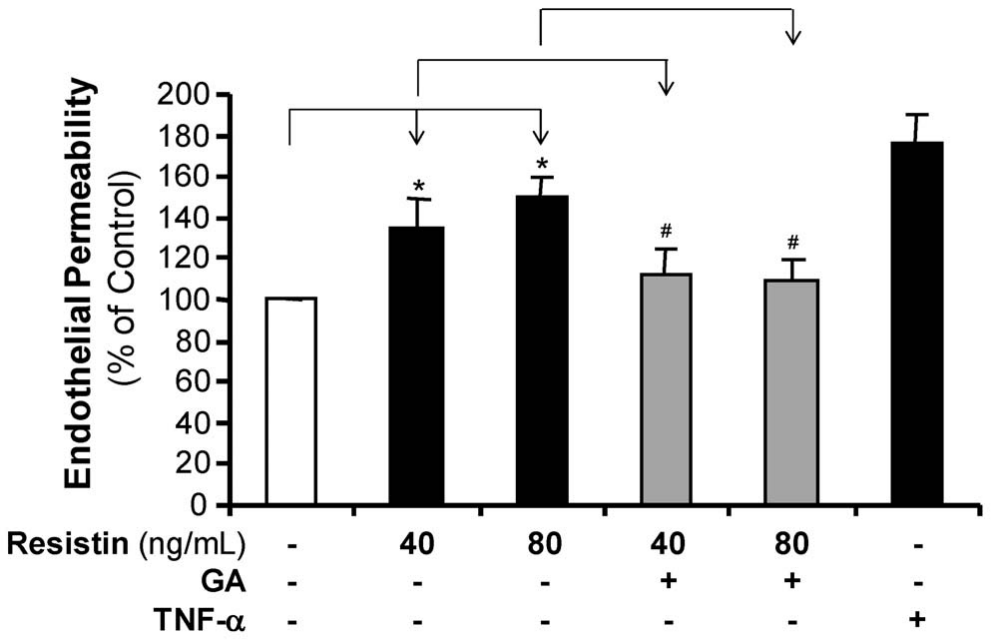
Effect of resistin on the permeability of HCAECs. Endothelial monolayer permeability was measured using the Costar Transwell permeability system with a fluorescence-labeled dextran tracer. HCAECs were treated with different concentrations of resistin (40 and 80 ng/mL), with or without Ginkgolide A (GA, 5 µM) pre-treatment, for 24 hours. Cells treated with TNF-α (2 ng/mL) served as a positive control. The results of the resistin-treated cells were compared with the results of control cells (n = 3, *P<0.05). The results of the cells that were pretreated with Ginkgolide A for 30 minutes before incubating them with resistin for 24 hours, were compared with results of the resistin-treated cells (n = 3, ^#^ *P*<0.05). The experiment was repeated thrice.

In order to determine the effect of resistin on the endothelial permeability, HCAEC monolayers were incubated with different concentrations of resistin and the permeability to Texas-Red-labeled dextran tracer was analyzed using a Costar Transwell system. The permeability of HCAEC monolayers treated with 40 or 80 ng/mL of resistin for 24 hours was 38% and 52% higher, respectively, than the permeability of untreated monolayers (*P*<0.05, Fig. 1). Monolayers treated with PBS or TNF-α(2 ng/mL) served as a negative or positive control, respectively [27]. Pretreating monolayers with Ginkgolide A (5 µM) for 24 hours effectively blocked resistin-induced permeability in HCAECs (*P*<0.05). By direct observation of cell morphology and cell density, current treatment of resistin did not cause cell death in HCAECs.

### Resistin Decreases the Expression of Junction Molecules in HCAECs

**Figure 2:**
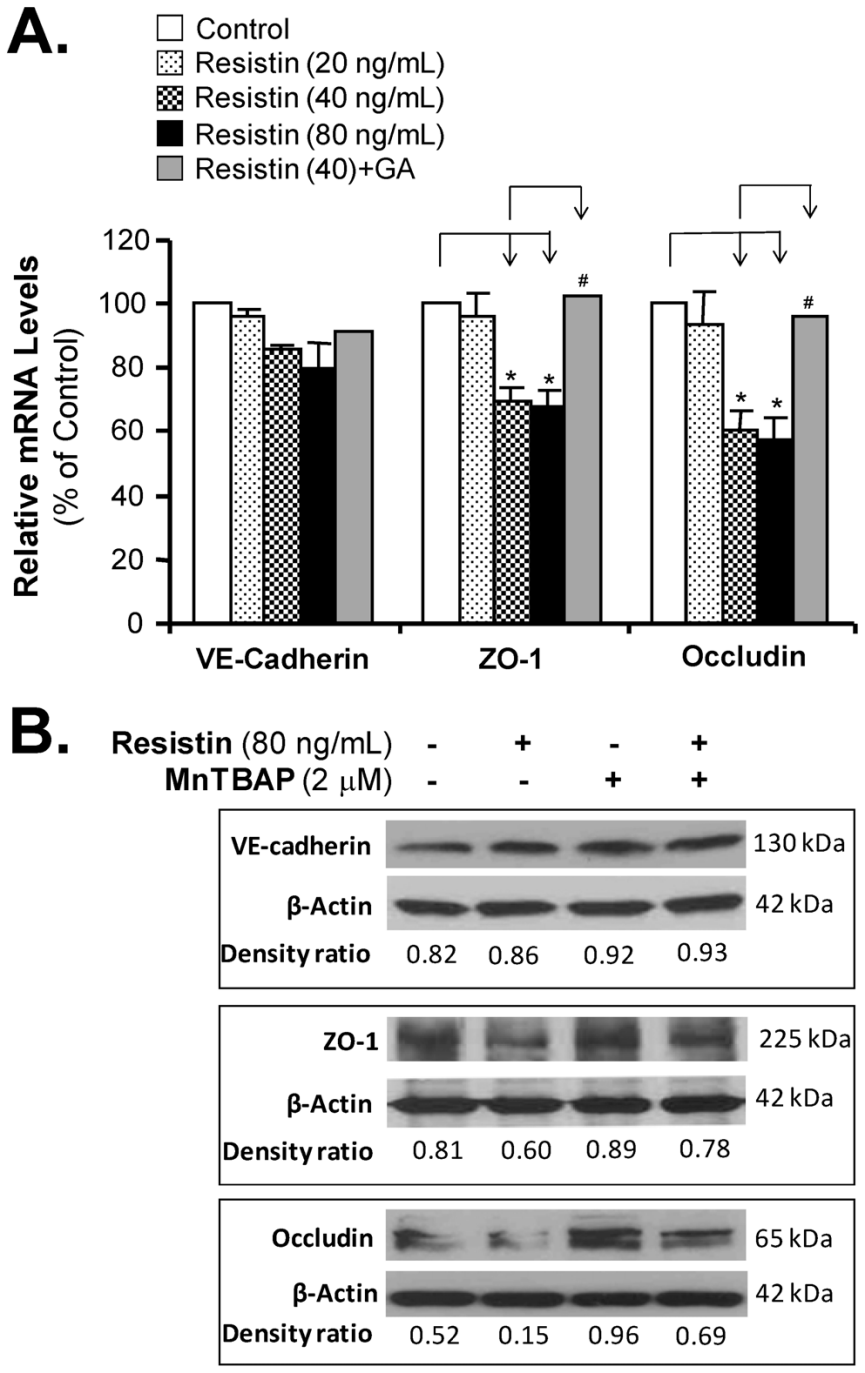
Effects of resistin on mRNA and protein levels of junctional molecules in HCAECs. (**A**)**.** HCAECs were treated with resistin (20, 40, and 80 ng/mL) for 24 hours, or pretreated with Ginkgolide A (5 µM) for 30 minutes before resistin treatment (40 ng/mL) for 24 hours. The mRNA levels of junction molecules (VE-cadherin, ZO-1, and occluding) were determined by real time PCR. The relative mRNA levels of each gene were normalized to the expression of a house keeping gene β-actin. The results of the resistin-treated cells were compared with the results of the control cells (n = 3, *P<0.05). The results of the cells that were pretreated with Ginkgolide A for 30 minutes and then treated with resistin (40 ng/mL) for 24 hours were compared with results of the resistin-treated cells (n = 3, ^#^ *P*<0.05). (**B**)**.** The protein levels of VE cadherin, ZO-1, and occludin were determined by Western blot analysis after resistin treatment and compared with controls. Equal loading control was monitored by reprobing the blot with anti-β-actin antibody. Western blot band density ratio for each tight junction protein and control β-actin was measured with ImageJ (1.47) software (NIH). To determine the effect of adding an antioxidant, cells were pretreated with MnTBAP (2 µM) for 30 minutes, and then incubated with resistin.

**Figure 3:**
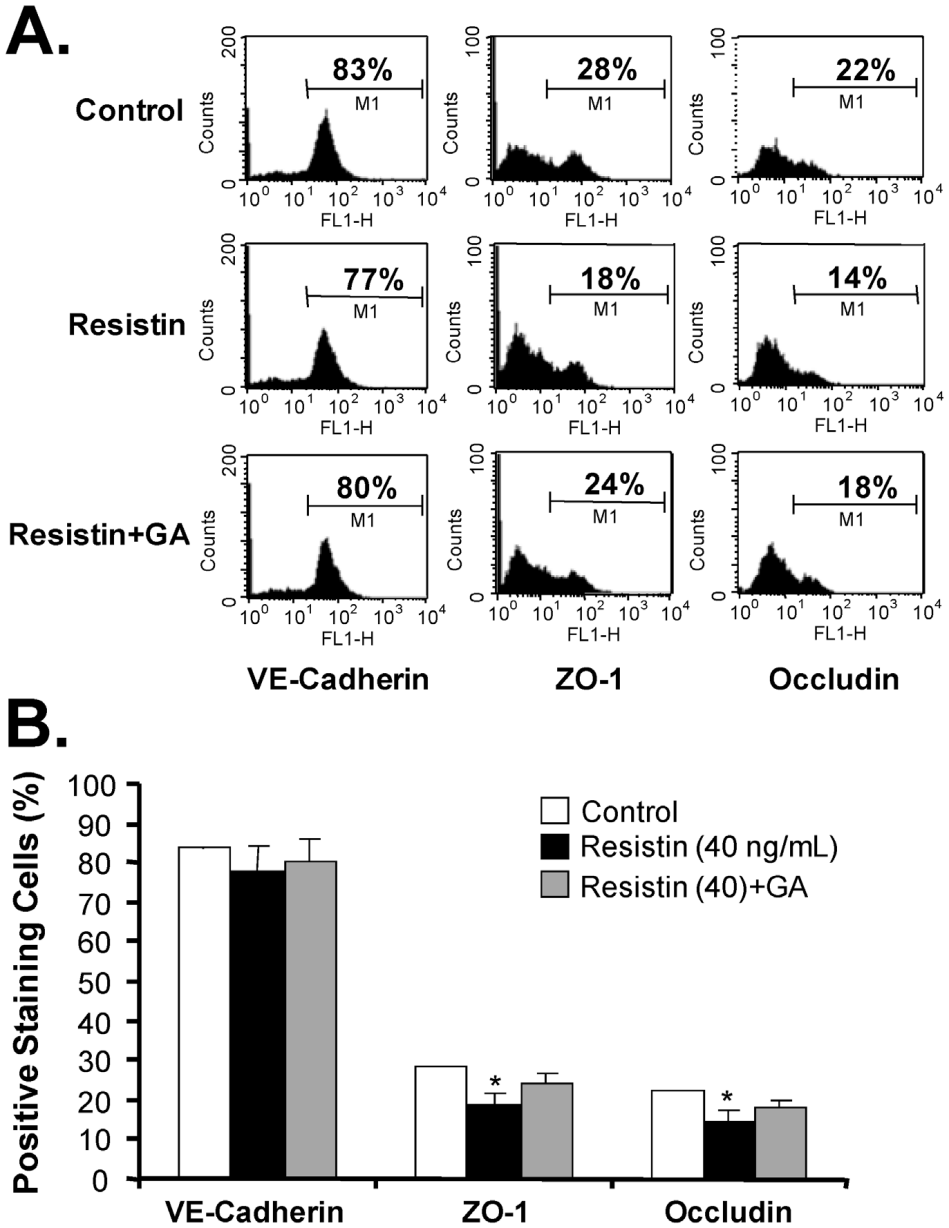
Flow cytometry analysis of junctional molecules in HCAECs. HCAECs were pretreated with Ginkgolide A (GA) for 30 minutes (or left untreated), and then treated with resistin (80 ng/mL) for 24 hours. The protein levels of junction molecules were determined by flow cytometry analysis. (**A**)**.** These representative histograms show the percentage of positively stained cells for each specific antibody against each junction protein, including VE-cadherin, ZO-1, and occludin. (**B**)**.** Bar diagram showing the average percentage of positively stained cells in three separate experiments. The results of the resistin-treated cells were compared with results of control cells (n = 3, *P<0.05).

To determine whether resistin could affect the endothelial junction structure, we determined the expression levels of two tight junction molecules (ZO-1 and occludin) and one adherens junction molecule (VE-cadherin) at the mRNA level using real time RT-PCR, and at the protein level using Western blot and flow cytometry analysis. HCAECs treated with resistin (40 ng/mL) had mRNA levels of ZO-1 and occludin that were 35% and 41% lower, respectively, than the corresponding levels in control cells (*P*<0.05, Fig. 2A). Western blot analysis was consistent with this result, showing that HCAECs treated with resistin (80 ng/mL) had a parallel decrease in the protein levels of ZO-1 and occludin (Fig. 2B). In addition, flow cytometry analysis revealed that the protein levels of ZO-1 and occludin in resistin-treated cells were 37% and 42% lower, respectively, than the corresponding levels in control cells (*P*<0.05, Fig. 3). There were no significant differences in the expression of VE-cadherin, either at the mRNA or protein level, between control and resistin-treated HCAECs. Furthermore, pretreatment of HCAECs with natural antioxidant Ginkgolide A (Fig. 2A and Fig. 3) or the commonly used antioxidant MnTBAP (2 µM) effectively blocked the resistin-induced downregulation of tight junction proteins ZO-1 and occludin (Fig. 2B).

### Resistin Increases ROS Production in HCAECs

**Figure 4:**
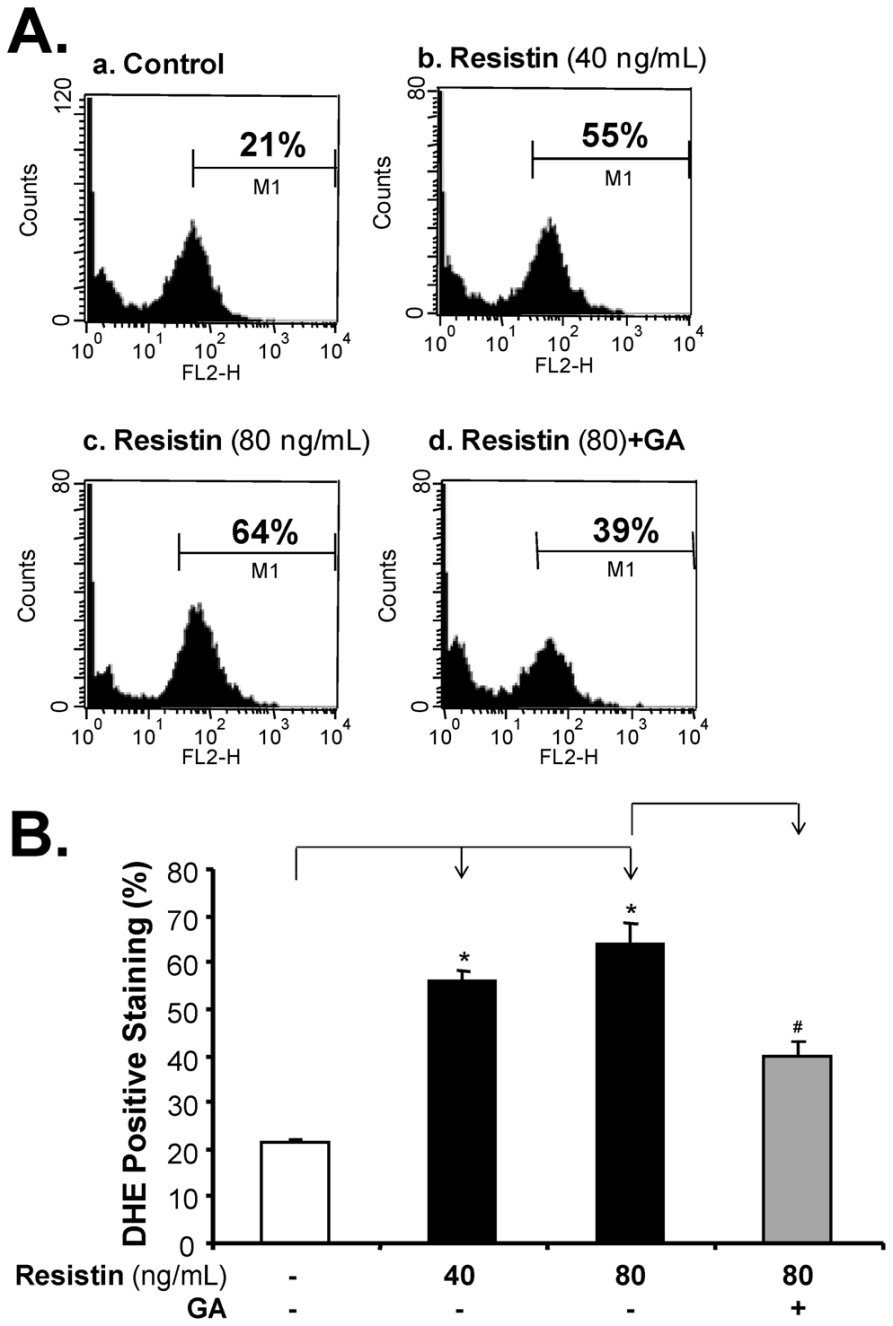
Effect of resistin on ROS production in HCAECs. Superoxide anion production was analyzed by DHE staining and flow cytometry analysis. (**A**)**.** Histogram showing DHE positively stained cells. (**a**). Normal HCAECs stained with DHE served as staining control. (**b, c,** and **d**) HCAECs were treated with different concentrations of resistin (40 and 80 ng/mL), or pretreated with Ginkgolide A and then incubated with resistin (80 ng/mL) for 24 hours, before staining with DHE (3 µM, 20 minutes). (**B**)**.** Bar diagram showing the average percentage of positively stained cells from three separate experiments. The results of the resistin-treated cells were compared with the results of control cells (n = 3, *P<0.05). The results of the cells pretreated with Ginkgolide A for 30 minutes followed by resistin treatment for 24 hours, were compared with the results of resistin-treated cells (n = 3, ^#^ *P*<0.05).

Reactive oxygen species (ROS), including superoxide anion (O_2_^−^), hydrogen peroxide (H_2_O_2_), hydroxyl radical (HO^−^), and peroxynitrite (ONOO^−^), may cause endothelial dysfunction [30], [31]. To investigate whether oxidative stress is involved in resistin-induced endothelial dysfunction, we analyzed the levels of O_2_^−^ using DHE staining and flow cytometry analysis. As shown in Fig. 4, incubating HCAECs with resistin (40 and 80 ng/mL) for 24 hours resulted in the cells significantly increasing O_2_^−^ production from 21% (control value) to 55% (cells treated with 40 ng/mL resistin), and from 21% to 64% (80 ng/mL resistin) (*P*<0.05). The natural antioxidant Ginkgolide A effectively blocked this resistin-induced increase in O_2_^−^ production in HCAECs. Thus, resistin-induced endothelial permeability in HCAEC is associated with an increase in O_2_^−^ production.

### Resistin Activates p38 MAPK in HCAECs

**Figure 5:**
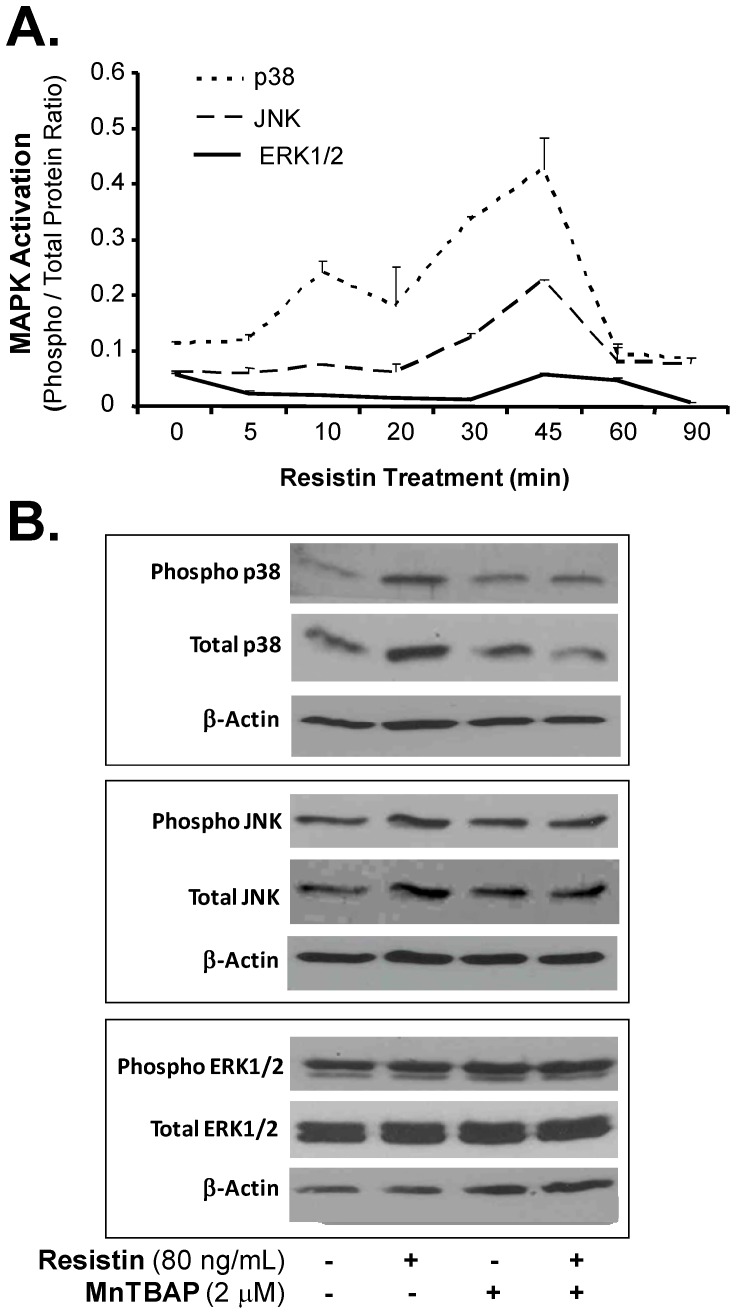
Effect of resistin on phosphorylation of MAPKs in HCAECs. (**A**)**.** The activation of MAPKs (ERK1/2, JNK, and p38) was determined using the Bio-Plex luminescence assay in HCAECs treated with resistin (80 µg/mL) for different time periods (0, 5, 10, 20, 30, 45, 60, and 90 minutes). The phosphorylated and the total protein for each MAPK were determined. (**B**)**.** HCAECs were incubated with or without resistin, or pretreated with MnTBAP before treating them with resistin (80 ng/mL) for 45 minutes. The protein levels of MAPK (total and phosphorylated proteins) were determined by Western Blot. β-actin was used as a loading control.

In order to understand the signaling pathway involved in resistin-induced permeability in HCAECs, we investigated the possible involvement of major MAPKs, including extracellular signal-regulated kinase (ERK1/2), p38, and the c-Jun N-terminal protein kinase (JNK). Bio-Plex luminescence immunoassay was used to make these determinations. The activation of MAPKs was studied by measuring the increase in MAPKs phosphorylation. Treating HCAECs with resistin (80 ng/mL) resulted in a substantial increase in the phosphorylation levels of p38 in these cells (peaked at 45 min of treatment) (Fig. 5A). A marginal increase of JNK phosphorylation was also observed (Fig. 5A). No substantial phosphorylation was observed for ERK1/2. To further confirm the activation of p38 MAPK protein, we analyzed whole cell lysates from untreated or resistin-treated HCAECs using Western blot. Western blot also confirmed the significant increase in p38 phosphorylation and the slight increase in JNK phosphorylation in resistin-treated HCAECs (Fig. 5B). Total p38 also increased in resistin-treated cells. Treating HCAECs with antioxidant MnTBAP for 30 minutes before incubating the cells with resistin (80 ng/mL) for 45 minutes completely blocked resistin-induced p38 activation. Thus, resistin activates the p38 signal transduction pathway through oxidative stress in HCAECs.

**Figure 6:**
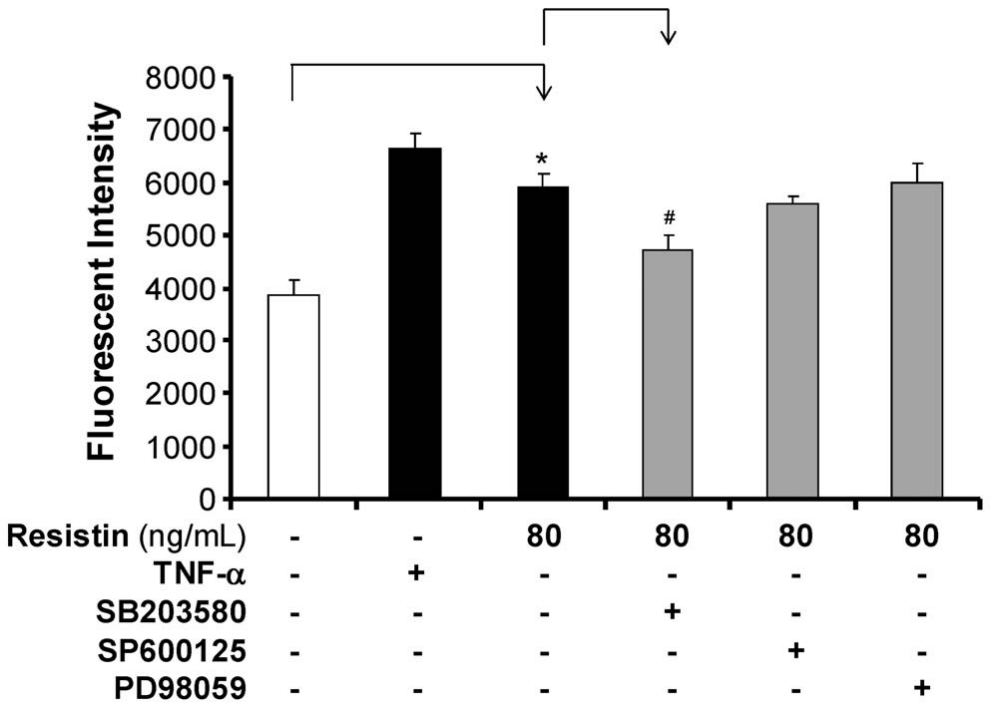
Effects of MAPK inhibitors on resistin-induced permeability in HCAECs. The effect of MAPK inhibitors on resistin-induced permeability in HCAECs was determined using a Transwell permeability assay. HCAECs were treated with resistin (80 ng/mL) alone, or pre-treated with each specific inhibitor of MAPKs (SB203580 for p38, SP600125 for JNK, and PD98059 for EEK1/2) for 30 minutes. Cells treated with TNF-α served as a positive control. The results of the resistin-treated cells were compared with the results of control cells (n = 3, *P<0.05). The results of the cells pretreated with MAPK inhibitor for 30 minutes, followed by resistin treatment for 24 hours,were compared with the results of the resistin-treated cells (n = 3, ^#^ *P*<0.05).

To confirm the role of MAPKs in resistin-induced endothelial permeability, HCAECs were incubated with specific inhibitors of either p38 (SB203580, 10 µM), JNK (SP600125, 25 µM), or ERK1/2 (PD098059, 50 µM), and then treated with resistin (80 ng/mL) for 24 hours. We chose the doses of the pharmaceutical inhibitors based on our previous experiments [28]. Endothelial permeability was determined using the Transwell system. As shown in Fig. 6, the permeability of HCAEC monolayers treated with resistin (80 ng/mL) was 53% higher than the permeability of control monolayers. When the monolayers were pretreated with p38 inhibitor, the effect of resistin was partially reversed. However, pre-treating the monolayers with JNK and ERK1/2 inhibitors had no effect on resistin-induced permeability. These results further suggest that resistin-induced permeability is mediated by the p38 signaling pathway.

## Discussion

In the present study, we demonstrate that treating HCAECs with resistin significantly increases the monolayer’s permeability *in vitro*. We propose that this effect is likely mediated by changes in the expression of tight junction molecules ZO-1 and occludin, whose levels decreased at both mRNA and protein levels in resistin-treated HCAECs. These effects are associated with an increase in ROS production and with p38 MAPK activation. Pretreating the monolayers with antioxidants (Ginkgolide A and SOD mimetic MnTBAP), or with the specific inhibitor of p38, can effectively block resistin-induced permeability in HCAECs. Our study demonstrates that resistin increases endothelial permeability, which may represent a critical link between resistin and atherosclerosis.

Endothelial cells control the passage of plasma constituents and circulating cells from blood to the underlying tissues. Therefore, maintaining the integrity of endothelial barrier functions is critical for maintaining normal physiological functions of the vascular system. Increased endothelial permeability contributes to pathological processes, such as inflammation, pulmonary edema, atherogenesis, and acute lung injury [14], [15], [17]. In this study, we used a Transwell permeability assay to determine the effect of resistin on HCAEC permeability. The Transwell cell culture model has been used successfully in this laboratory to analyze the effects of ritonavir [32], lysophosphatidylcholine [29], secretoneurin [28], stanniocalcin-1 [27], efavirenz [33], and eotaxin [34] on endothelial permeability *in vitro.* Our data from the current study show that clinically relevant concentrations of resistin can significantly increase monolayer permeability in cultured HCAECs. The permeability of HCAEC monolayers treated with 40 and 80 ng/mL of resistin was 38% and 50% higher, respectively, than the permeability of control monolayers. Hogan *et al.* found that RELM-β is predominantly expressed by goblet cells and colonic epithelial cells and is involved in the maintenance of colonic epithelial cell barrier function [35]. The phenomenon of resistin-induced permeability has also been observed in human umbilical vein endothelial cells (HUVECs). For example, Langheim *et al.* [36] found that high concentrations of resistin generated in the conditional media from the epicardial adipose tissue (EAT) of acute coronary syndrome (ACS) patients significantly increased para-endothelial transit of albumin through HUVEC monolayers *in vitro*. This finding suggests that resistin is a major inducer of endothelial damage through the induction of permeability.

The integrity of endothelial junction proteins, including transmembrane tight junction proteins (occludin, claudin, and JAM-1), transmembrane adherens junction protein (VE-cadherin), and intracellular proteins (ZO-1, ZO-2, and ZO-3), is critical for maintaining endothelial barrier functions and permeability [37]–[42]. Any alteration of these junctional protein structures could increase endothelial permeability causing endothelial dysfunction, cell proliferation, transformation, metastasis, and cancer [43]. In our study, we demonstrate that resistin treatment significantly down-regulates the expression of endothelial junction proteins, particularly tight junction proteins ZO-1 and occludin, at both mRNA and protein levels. Although expression of VE-cadherin was slightly decreased in response to resistin, the change was not statistically significant. These results indicate that down-regulation of tight junction proteins may be a key mechanism of resistin-mediated paracellular permeability increases in endothelial cells. Reduced numbers of tight junction proteins may lead to further damage to the vascular wall and the endothelial monolayer, thus accelerating the development of atherosclerosis.

Oxidative stress occurs when the production of free radicals in the human body exceeds the body’s ability to neutralize and eliminate them. Oxidative stress can result from a deficiency of antioxidants or from over-production of free radicals. Excessive generation of ROS and reactive nitrogen species (RNS) by activated neutrophils and endothelial cells has been implicated in the pathophysiology of endothelial barrier dysfunction [44]–[46]. Disruption of the integrity of this barrier markedly increases permeability to fluids, solutes, and inflammatory cells, and is the hallmark of many disorders such as acute lung injury (ALI) and sepsis. The effects of oxidative stress on the endothelium of the vascular wall include direct oxidation and nitration of cytoskeleton proteins, such as actin and tubulin, and indirect modulation of kinase pathways, such as protein kinase C, ERK, Src, and Rho [47], [48]. ROS can not only quench NO activities through chemical reactions, but also alter the regulation of endothelial nitric oxide synthase expression, which in turn causes NO-related functional permeability changes in endothelial cells [49], [50]. In the present study, we demonstrate that treating HCAECs with resistin results in the cells increasing their production of superoxide anion, in a concentration-dependent manner. These findings suggest that resistin regulates monolayer permeability through increased oxidative stress. This possibility is further supported by the effects of two different antioxidants in our study. The natural antioxidant Ginkgolide A blocked resistin-induced permeability in HCAEC monolayers. Meanwhile, the SOD mimetic MnTBAP effectively blocked the resistin-induced decrease in the levels of tight junction proteins ZO-1 and occludin, as well as the level of p38 MAPK activation. The use of antioxidants may be an effective strategy in preventing resistin-induced endothelial dysfunction.

MAPKs (JNK, ERK1/2, and p38) are serine-threonine kinases that perform important functions mediating cellular responses to a variety of extracellular stimuli and are reported to be involved in endothelial cell activation and cardiovascular disease [51]–[55]. To investigate the involvement of MAPK signal transduction molecules in resistin-induced permeability in HCAECs, we checked the activation of MAPKs using Western blot and Bio-plex luminuoassay. We demonstrate that treating HCAECs with resistin induces activation of p38 and, to a lesser extent, of JNK, but not ERK1/2. To further confirm the critical role of p38 and JNK activation in resistin-induced permeability in HCAECs, we preincubated the cells with specific inhibitors of p38 and JNK. The p38 inhibitor SB203580 was able to effectively block the resistin-induced permeability in HCAECs. MAPK p38 has been shown to play an important role in the *Staphylococcus aureus* induced lung micro and macrovascular barrier dysfunction [56]. However, this effect was absent with JNK inhibitor SP600125. Thus, the molecular mechanisms underlying the increase in permeability induced by resistin in HCAECs include the activation of the p38 signaling pathway and increased oxidative stress. These findings are consistent with those in our previous publication [57].

**Figure 7:**
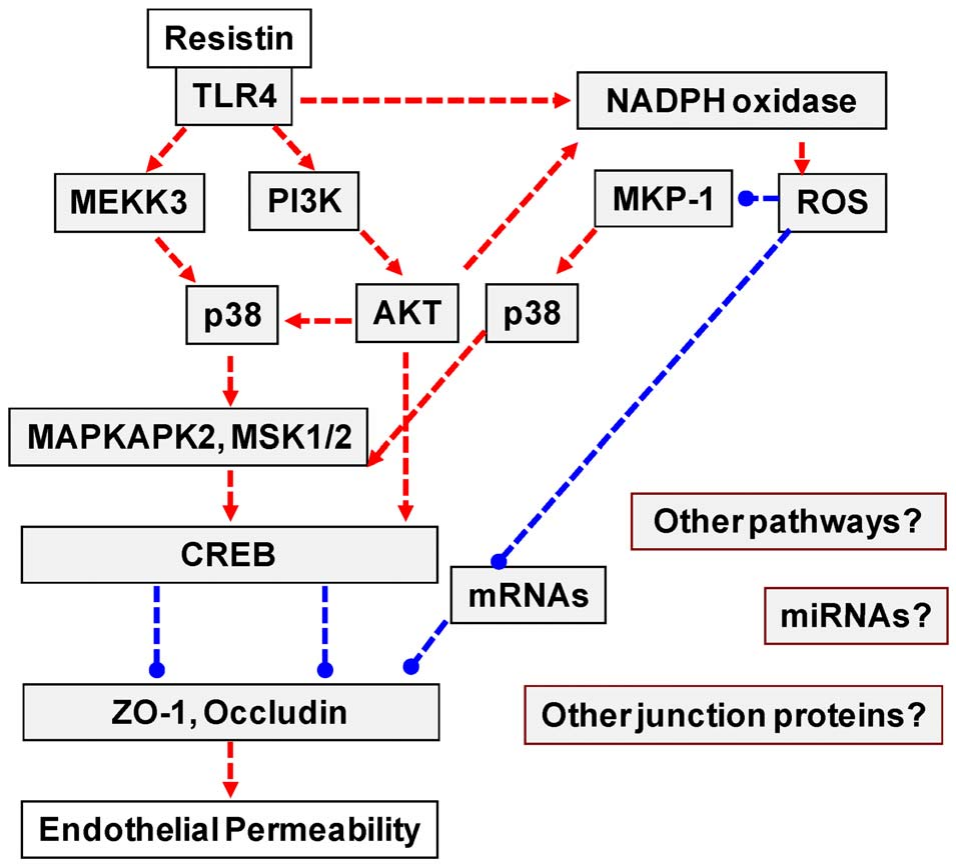
Potential molecular mechanisms (hypothesis) of resistin-induced endothelial permeability. Resistin may interact with toll-like receptor 4 (TLR4), inducing a TLR4-mediated signaling cascade such as the activation of p38, NADPH oxidase and transcriptional factor CREB, which directly and/or indirectly reduces the expression of ZO-1 and occludin at transcriptional and/or post-transcriptional levels.

**Figure 8:**
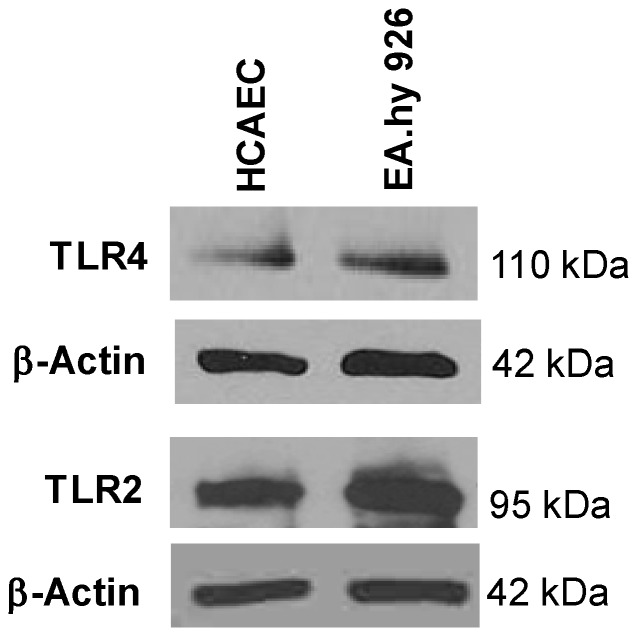
Expression of TLR4 and TLR2 in human endothelial cells. HCAECs and EA.hy926 (a human umbilical vein cell line) were lyzed with cell lysis buffer. Protein levels of TLR4 and TLR2 were determined by Western blot. β-actin was used a loading control.

Although we have shown in this study that resistin increases endothelial permeability through activation of p38 MAPK, oxidative stress and downregulation of ZO-1 and occludin, the detailed regulation mechanisms among these molecules in endothelial cells are not well understood. Considering current data and other data from literatures, we hypothesize that resistin may interact with toll-like receptor 4 (TLR4), inducing a TLR4-mediated signaling cascade such as the activation of p38, NADPH oxidase and transcriptional factor CREB, which directly and/or indirectly reduces the expression of ZO-1 and occludin at transcriptional and/or post-transcriptional levels (Fig. 7). A recent report indicates that resistin may bind to TLR4, but not TLR2, on human myeloid and epithelial cells and regulate cytokine expression [58]. We have confirmed that human endothelial cells do express TLR4 and TLR2 (Fig. 8). Mammalian TLR4 is a signal-transducing receptor activated by bacterial lipopolysaccharide (LPS) and high-mobility group protein B1 (HMGB1) as well as other ligands [59]. LPS interacts with TLR4 and induces the activation of p38 by different pathways including MEKK3, PI3K-AKT, and ROS-MKP-1 axis [60]–[62]. Activated AKT can directly phosphorylate p47phox [63] and Rac1 [64], [65] to activate NADPH oxidase. LPS treatment is known to induce p47phox phosphorylation by IRAK4 [66]. ROS can activate p38 by oxidation of several phosphatases such as MKP-1 at their active site cysteine residues that would otherwise inhibit the p38 pathway [67], [68]. CREB is activated by phosphorylation at Ser133 by various signaling pathways including p38 and AKT [69]. p38 has been shown not to directly phosphorylate CREB; however, activation of its downstream substrates MAPKAPK2 and MSK1/2 are known to be responsible for the phosphorylation of CREB [70]–[73]. Recent studies indicate CREB directly involves in gene expression of ZO-1 at the transcriptional level [74]. ZO-1 promoter contains several potential CREB binding sites [75]. In addition, ROS could increase the degradation rate of mRNAs of these proteins [76]. Moreover, resistin could induce different signaling pathways and affect the expression of microRNAs and other junction proteins, which could contribute to the increase in endothelial permeability. Further investigation to confirm the hypothesis described above in endothelial cells is warranted.

Taken together, our data provide a clear link between resistin and *in vitro* endothelial permeability. We propose that the underlying molecular mechanisms include down-regulation of endothelial junction proteins, increased oxidative stress, and activation of p38 MAPK. A deeper understanding of the specific molecular mechanisms and pathways implicated in resistin-mediated endothelial dysfunction remains a fertile area for further research, and the opportunity to intervene early in the process of endothelial dysfunction leading to vascular disease represents an exciting therapeutic possibility.
